# A Comparative Evaluation of the Efficacy of Two Novel Desensitising Dentifrices

**DOI:** 10.1155/2012/896143

**Published:** 2012-12-06

**Authors:** Ashley P. Barlow, Jane He, Cindy Tian, Peter Jeffery, Stephen C. Mason, Bao-Jun Tai, Han Jiang, Gareth D. Rees, Min Quan Du

**Affiliations:** ^1^GlaxoSmithKline Consumer Healthcare, Surrey Weybridge, KT13 0DE, UK; ^2^Sino-American Tianjin SmithKline and French Laboratories Ltd., Beijing, China; ^3^Key Laboratory of Oral Biomedicine Ministry of Education, School & Hospital of Stomatology, Wuhan University, LuoYu Road 237, Wuhan 430070, China; ^4^Genesis Oral Bioscience, Consultancy & Scientific Writing, Regents Mews, Surrey, Horley RH6 7AN, UK

## Abstract

A comparison of the desensitising efficacy of two commercially available dentifrices with different modes of action was conducted in a randomised, examiner-blind, two-arm, parallel group, 8-week, longitudinal clinical study. Dentifrice A, (Sensodyne Multi Action Iso-Active), contained 50000 ppm KNO_3_ and 1450 ppm fluoride as NaF. Dentifrice B, Colgate Sensitive Pro-Relief, contained a combination of 80000 ppm arginine, bicarbonate, calcium carbonate, and 1450 ppm fluorine as NaMFP. Subjects (*N* = 110), stratified into two groups (*N* = 55), brushed twice-daily for 60 s, over an 8-week period. Sensitivity status, compliance, and safety were determined at 1, 2, 4, and 8 weeks. A fixed-effects ANCOVA statistical model was applied to the Intent-To-Treat population using a two-sided 5% significance level. After 8 weeks, the treatment groups using Dentifrice A and Dentifrice B exhibited mean reductions from baseline of 49% and 45% in air sensitivity visual analogue scale (VAS) score, 61% (both) in examiner-based Schiff Sensitivity score, and clinically significant reductions in tactile pain threshold; all reductions were statistically significant (*P* < 0.0001). Both treatment groups also exhibited significant reductions across all sensitivity measures at 1, 2, and 4 weeks (*P* ≤ 0.0059, Dentifrice A; *P* ≤ 0.0137, Dentifrice B).

## 1. Introduction

Dentine hypersensitivity is a common condition variously reported to affect anywhere between 3 and 57% of the population depending on the method of diagnosis, geography, and population chosen [1–4]. It is characterised by a short sharp pain arising from exposed dentine in response to thermal, evaporative, tactile, osmotic, or chemical stimuli that cannot be ascribed to any other form of dental defect [4, 5]. Although symptoms of the condition are acute and episodic they can persist for years. Without proper clinical management dentine hypersensitivity can have a significant impact on a sufferers quality of life [6, 7]. Human dentine contains large numbers of fluid-filled tubules, typically 1–5 *μ*m in diameter, that run radially from the dentine-pulp junction to the surface of the dentine-enamel or dentine-cementum junction [8, 9]. Tubule lumens, that are unsclerosed, free of debris, and open at the dentine surface, facilitate transmission of the stimulus to the pain receptors present at the pulp/odontoblast interface [10]. As a consequence, a key micromorphological factor influencing the severity of the sensitivity response is the extent to which the dentine surface and thus the tubules are occluded by a smear layer [10]. The prevailing mechanistic description of stimulus transduction is the hydrodynamic theory elucidated by Brannström over 40 years ago [11–13]. In the event that externally applied stimuli significantly alter fluid flow within the dentinal tubule, this may be sufficient to trigger mechanoreceptors near the base of the tubule and firing of afferent nerves [10]. Aetiological factors relevant to the development of dentine hypersensitivity include erosive wear close to the gingival margin where the enamel is thinnest, its removal resulting in exposure of the underlying dentine. Gingival recession resulting from periodontal disease or tooth brushing trauma has also been considered to be an aetiological factor in dentine hypersensitivity as this may result in the exposure of the tooth root and associated cementum. Relative to enamel, the cementum is more susceptible to removal through erosive wear, a process that rapidly leads to exposure of the underlying dentine [14, 15].

An implicit consequence of the hydrodynamic theory is the polarisation of treatment options into those that target desensitisation of the relevant intradental nerves, and those whose aim is to inhibit or prevent transmission of the stimulus itself. Potassium salts including nitrate, chloride, and citrate are examples of the former, with dentifrice being the principal delivery format [16, 17]. Although clinical efficacy has been widely reported by Hodosh and others [16–19], and the ability of potassium ions to suppress nerve firing demonstrated [20], the precise mode of action remains elusive [19–22]. The second strategy for the treatment of dentine hypersensitivity is to employ occlusion agents to seal or at least partially occlude the patent tubules; this may also be achieved through formation of a barrier layer on the surface of the dentine. Although considered a nonphysiological approach, such treatments often rely on the reactivity of the desensitising agent to promote formation of mixed mineral deposits on and within the exposed dentine [22, 23]. Examples that have been widely reported to be effective *in vivo* include oxalate salts [24–26] and strontium salts [27–29]. Their occlusion efficacy *in vitro* has also been widely studied using techniques that include measurement of dentine permeability [30, 31], scanning electron microscopy [28, 32], and radiopacity [26]. The utility of polymer-based sealants as occlusion agents for treating dentine hypersensitivity has also been investigated [33, 34].

A new occlusion-based technology has recently been commercialised in a dentifrice format for the treatment of dentine hypersensitivity [35]. Based on arginine, bicarbonate, and calcium carbonate, its utility both as an anticaries and desensitising active has previously been claimed [36, 37]. In the latter case, the technology is reported to work through binding of positively charged agglomerates to exposed dentine surfaces and within the tubules themselves [38]. The marketed dentifrice contains fluorine as sodium monofluorophosphate (NaMFP), and its desensitising efficacy has been reported in a comparative clinical study against a dentifrice containing the safe and effective monographed desensitising agent potassium nitrate (KNO_3_) as the benchmark control [39, 40]. As well as providing oral health benefits, a dentifrice should ideally meet basic aesthetic criteria such as acceptable taste and mouth feel, since this may affect compliance and consequently efficacy [41]. It is relevant to note, therefore, that the recent development of gel-to-foam dentifrices wherein a volatile agent is incorporated into the formulation, that the foaming *in vivo* is significantly enhanced [42]. A recently published clinical study employing a desensitising gel-to-foam dentifrice containing KNO_3_ reported highly significant relief from tooth sensitivity at 4, 8, and 12 weeks [43]. The improved *in vivo* dispersion associated with this novel format is believed to enhance delivery of the oral care actives in hard-to-reach areas [43]. The aim of the present study was to compare the effectiveness of the two aforementioned dentifrices in an 8-week, two-arm, parallel group, longitudinal dentine hypersensitivity study.

## 2. Materials and Methods

### 2.1. Materials

Sensodyne Multi Action Iso-Active (Dentifrice A) contained 50000 ppm KNO_3_ and 1450 ppm fluoride as the sodium salt (NaF) and was manufactured by GlaxoSmithKline Consumer Healthcare (Brentford, Middlesex, UK). Colgate Sensitive Pro-Relief (Dentifrice B) contained 80000 ppm arginine, bicarbonate, calcium carbonate, and 1450 ppm fluorine as NaMFP and was manufactured by Colgate-Palmolive (Guildford, Surrey, UK). Oral B Indicator Soft toothbrushes were sourced from Procter & Gamble (Weybridge, Surrey, UK). Test dentifrices were supplied to study volunteers in their original commercial packaging over-wrapped in opaque vinyl with a study-specific label detailing protocol number, product code, storage conditions, and precautionary information including an emergency contact telephone number.

### 2.2. Ethical Aspects, Inclusion Criteria, Efficacy Assessment, and Randomisation

The study protocol was reviewed and approved by an Independent Ethics Committee at Wuhan University, China and conducted according to ICH GCP guidelines [44]. Inclusion criteria required volunteers to be aged between 20 and 60 years of age, with at least 20 natural permanent teeth and pre existing self-reported and clinically diagnosed tooth sensitivity. Subjects were required to present with test teeth exhibiting signs of facial/cervical erosion and/or abrasion and/or gingival recession. Test teeth should, in the opinion of the examiner, otherwise exhibit good gingival health and should not exhibit any clinical mobility (mobility score < 1) [45]. Subjects must have successfully completed a visual analogue scale (VAS) instruction exercise at screening, whose purpose was to train subjects in the use of VAS by requiring subjects to estimate how much of the total area of a series of 7 shapes had been shaded using a 100 mm line anchored at one end with “No Shading” and the other with “Complete Shading” [46]. Subjects were excluded if they had received desensitising treatments or used a desensitising dentifrice within the previous 3 months.

Tactile and air sensitivity measurements were performed by two independent examiners. At the screening visit, the tactile sensitivity of each eligible tooth was determined using a calibrated Yeaple probe (XiniX Research Inc., Portsmouth, NH, USA) set at the equivalent microamperes to deliver a fixed force of 30 g. The probe tip was placed perpendicular to the buccal surface and moved in a slow distal-to-medial sweeping motion across the tooth surface in order to ensure application of the stimulus across the sensitive area of the exposed dentine. Subjects were then asked whether the sensation was painful, with responses confined to “yes” or “no”. Subsequent testing at weeks 1, 2, 4, and 8 was performed by a single examiner on the two teeth selected at baseline. Testing began at 10 g and increased by 10 g with each successive challenge until a “yes” response was obtained or the 50 g upper limit reached. The force setting eliciting the positive response was then repeated, but in the absence of a second positive response the force setting was increased by 10 g and continued until a force was found that elicited two consecutive “yes” responses.

The evaporative (air) assessment was taken after the determination of tactile sensitivity with a minimum of 10 min recovery time between each evaluation. It was felt that 10 mins would allow sufficient time for physiological and neurological recovery following the first set of stimuli. The test involved directing an air stream (60 ± 5 psi) at ambient temperature for 1 s from a triple air dental syringe toward the exposed dentine surface from a distance of approximately 1 cm; adjacent teeth were shielded from the stimulus by the fingers of the examiner. Each tooth was scored according to the four-point Schiff Air Sensitivity Scale [47], defined as follows.

0 Subject does not respond to air stimulus. 

1 Subject responds to air stimulus, but does not request discontinuation of stimulus.

2 Subject responds to air stimulus and requests discontinuation or moves from stimulus.

3 Subject responds to air stimulus, considers stimulus to be painful, and requests discontinuation of the stimulus.

At the screening visit, the teeth of subjects eliciting a score of 2 or 3 were recorded as qualifying as potential test teeth. 

At the baseline visit, subjects were also asked to rate the pain intensity associated with the evaporative (air) stimulus using a linear 100 mm VAS end-anchored by “No Pain” and “Worst Pain Imaginable”. At least two incisors, canines, or premolars were required to provide an air sensitivity response of ≥25 mm based on the VAS, a Schiff score ≥ 2, and a tactile pain threshold between 10 and 50 g. Subject randomisation was performed using as stratification factors; the number of air sensitive teeth at baseline based on the VAS response (<6/≥6) and the mean VAS score at baseline (<60 mm/≥60 mm). In total, 63 (57.3%) of subjects had 6 or more sensitive teeth at baseline, 84 (76.4%) had a mean baseline VAS score ≥ 60 mm. A total of 110 eligible adult volunteers took part in this study after signing an informed written consent form. Of these, 19 (17.3%) were male and 91 (82.7%) were female, with a mean (±SD) age of 42.0 ± 10.43 years; all subjects were of Chinese ethnicity. 

### 2.3. Study Design

This was an 8-week, single-centre, examiner-blind, randomised, two-arm, parallel group study in otherwise healthy subjects experiencing dentine hypersensitivity. The clinical trial conformed to consensus design and conduct recommendations regarding population selection, sample size, test stimuli, assessment, and outcome variables [5]. Eligible subjects were randomised to the two treatment groups (*N* = 55 per treatment group). Two test teeth were selected on the basis of their eligibility criteria for both tactile and evaporative stimuli. Subjects were given their assigned dentifrice and toothbrush to use in place of their regular oral hygiene products for the next 8 weeks. Subjects were not permitted to use any oral care products after baseline other than the dentifrice and toothbrush provided. Prior to the baseline visit, subjects were instructed to refrain from oral hygiene procedures and gum chewing for a minimum of 8 h before their scheduled appointment. Subjects were reminded not to eat or drink for a minimum of 2 h nor take analgesics for at least 8 h prior to their scheduled appointments. Dental scaling, prophylaxis, and bleaching were prohibited for the duration of the study.

The test product was used for one-timed minute twice per day (am/pm) with subjects instructed to dispense a ribbon of paste to cover at least three quarters of the head of the toothbrush. During the treatment phase, subjects returned to the study site after 1, 2, 4 and 8 weeks following their baseline visit for evaluation of dentine hypersensitivity, oral health status, and continuing eligibility. Test products were weighed at each visit as a means of monitoring compliance. The primary efficacy measure was observance of a statistically significant within treatment reduction from baseline in the subjects' assessment of air sensitivity, measured using the VAS, after 8 weeks. Secondary efficacy measures included changes in Schiff score and tactile pain threshold after 8 weeks. The definitions of reduction in sensitivity in each case are a decrease of at least 10 mm in mean VAS score, a decrease in mean Schiff score across the two teeth of at least 0.5 units, and a mean increase of at least 10 g in the tactile pain threshold determined with the Yeaple probe. The safety profile of both dentifrices was assessed by reference to adverse events and oral soft tissue abnormalities.

### 2.4. Statistical Analyses

The subject level change from baseline was calculated as the mean change from baseline observed across the two selected teeth within a subject. A fixed effects analysis of covariance (ANCOVA) model was used with treatment included as a fixed effect factors and baseline number of air sensitive teeth and mean baseline VAS score across the two teeth included as covariates. All significance tests were two-sided and performed at the 5% level of significance with no adjustments for multiple comparisons.

For analyses of subject level Schiff data and subject level Tactile Pain Threshold data, an ANCOVA model was used with treatment and baseline VAS stratification value and included as fixed effect factors. Baseline number of air sensitive teeth and either mean baseline Schiff score or mean baseline Tactile Pain Threshold score across the two teeth were included as covariates, respectively. Intent-To-Treat (ITT) and Per Protocol (PP) analyses were performed for within and between treatment assessments of change from baseline to weeks 1, 2, 4, and 8. Two-sided significance tests were performed at the 5% level with no adjustments for multiple comparisons.

Tooth level analyses were also performed wherein each tooth individually contributed to the analyses, as opposed to the mean across the two teeth used for the subject level analyses. The statistical models were similar to those used for subject level data, but with the addition of subject included as a random effect. ITT analyses were performed for within and between treatment assessments of change from baseline to weeks 1, 2, 4, and 8. Two-sided significance tests were performed at the 5% level with no adjustments for multiple comparisons.

## 3. Results

The primary population for the analysis of all efficacy data was the ITT population. One subject withdrew from the study and provided no postbaseline efficacy data leading to an ITT population of 109 subjects. One subject who received antibiotics for gingival inflammation was excluded yielding a PP population of 108 subjects. Other major protocol violations were reported for a further 13 subjects leading to the exclusion of specific data from PP analyses but not full exclusion of subjects. Specifically, 8 subjects exhibited noncompliance with the study product, 3 were significantly noncompliant with the visit schedule and 2 subjects used a nonstudy toothbrush.

A statistically significant reduction from baseline in tooth sensitivity was observed for both test products, for all three sensitivity parameters, at all four time points, including the primary analysis of sensitivity reduction at week 8 based on mean VAS score as shown in Table 1. There were no statistically significant differences between the two study products in the reduction of sensitivity from baseline to any of the four time points, for any of the three sensitivity parameters, as shown in Table 2. The number and percentage of subjects experiencing a reduction in sensitivity from baseline after 8 weeks of treatment is summarised in Table 3 for each sensitivity measure. Of those subjects treated with Dentifrice A, 50 (93%) exhibited a reduction in air sensitivity measured using the VAS, 53 (98%) exhibited a reduction in air sensitivity based on Schiff score, and 47 (87%) exhibited a reduction in tactile sensitivity based on force-pain threshold. The equivalent numbers for subjects treated with Dentifrice B were 44 (88%) for the VAS, 49 (98%) for Schiff score, and 42 (84%) for the tactile pain threshold. 

The PP analyses performed on subject level data and additional ITT analyses performed on tooth level data of within and between treatment outcomes yielded comparable results to those obtained using the primary subject level ITT approach (data not shown). The sole exception related to the PP analysis of VAS score, where the reduction from baseline at 1 week for the treatment group using Dentifrice B was not statistically significant (*P* = 0.0645). Investigation of the relationship between efficacy variables yielded correlation coefficients of 0.74, −0.55, and −0.53, respectively, for VAS score versus Schiff score, Schiff score versus tactile pain threshold, and VAS score versus tactile pain threshold. 

Twelve subjects reported a total of 19 treatment emergent adverse events of which 11 were related to oral health status; 3 were associated with the treatment group using Dentifrice A and 8 with the group using Dentifrice B. Two adverse events were judged to be possibly related to the study treatment (Dentifrice B) and were reported by the same subject, however both were mild in intensity and resolved at study completion. No serious adverse events were reported during the course of this clinical study.

## 4. Discussion

In this study, the efficacy of two new desensitising dentifrice formulations has been compared in a blinded, parallel group, controlled clinical study. The two dentifrices are very different both in terms of composition and their claimed mode of action. Dentifrice A contains KNO_3_, a proven nerve desensitising active, and NaF as the source of ionic fluoride. The formulation also contains 2-methyl butane, a nonsurfactant-based foaming agent with a boiling point of *ca.* 30°C that is activated *in situ* on brushing. Dentifrice B, in contrast, contains a novel dentine occlusion system that includes the dibasic amino acid arginine, bicarbonate, and calcium carbonate. The formulation does not contain fluoride due to the presence of calcium, but contains fluorine as NaMFP in order to confer anticaries activity.

The results demonstrate comparable clinical effectiveness for the two test dentifrices with significant reductions in measures of tooth sensitivity observed across all measures, at all predefined time points. After 8 weeks of twice daily use, a 49% versus 45% reduction in the subjective outcome measure of evaporative VAS score, and a 61% versus 61% reduction in examiner-based Schiff score was observed for Dentifrice A and B, respectively, versus baseline. Large and clinically relevant improvements in the tactile pain threshold versus baseline were also observed for the two treatment groups. No significant between-treatment efficacy differences were apparent at any of the time points. Given the comprehensive improvements in sensitivity relief in both treatment groups across all the efficacy measures, these results suggest a comparable level of performance between the test products with no apparent difference between the potassium-based novel gel-to-foam formulation (Dentifrice A) and the arginine-based formulation (Dentifrice B). Tooth-level analyses revealed one minor difference to the subject-level analysis wherein the evaporative VAS score in the treatment group using the arginine-based dentifrice was not statistically different from baseline after 1 week of treatment (*P* = 0.0645). Given the number of the statistical evaluations conducted, this specific outcome does not materially change the overall inferences drawn from these results of this clinical study. The correlation coefficients used to examine the relationship between the different efficacy variables showed a moderately strong correlation between the VAS score and Schiff score (0.78). This is not unexpected given that the data contributing to both are elicited by the same evaporative air stimulus. Comparisons of the Schiff score versus tactile pain threshold and VAS score versus tactile pain threshold elicited similar correlation coefficients (−0.55 and −0.53, resp.). That they are less strongly correlated than the VAS and Schiff scores is again to be expected.

As previously indicated, two recently published 8-week dentine hypersensitivity studies reported superior efficacy for the occlusion-based Dentifrice B containing the novel arginine/bicarbonate/calcium carbonate technology when compared with a conventional desensitising dentifrice containing KNO_3_ [39, 40]. In the aforementioned study, efficacy was determined using two outcome measures, mean Schiff score, and mean tactile pain threshold and were found to be statistically superior for the arginine-based dentifrice at 2, 4, and 8 weeks (*P* < 0.05). The present study employed a similar clinical methodology that accords with published consensus recommendations [5], albeit the authors acknowledge that a negative control arm would have helped to contextualise the extent of the efficacy benefit offered by either of the treatments. The lack of negative control does make it is problematic to interpret the clinical relevance of the response measured in either treatment group in this specific study. Future clinical studies on either product should look to incorporate a negative control arm to address this gap in the scientific design. Nonetheless, the sample size employed in this study was substantially higher (*ca.* 38%) in comparison to the previously cited studies involving Dentifrice B [39, 40], and additionally employed a subjective patient-assessed measure of product performance via the VAS score. As such the current study was adequately powered to differentiate any clinically relevant differences in product performance. The results in the present study demonstrate no difference in efficacy for the two dentifrices in respect of the observed reductions of in self- and examiner-assessed dentine hypersensitivity at all observed time points (1, 2, 4, and 8 weeks). These two novel formulations were also well tolerated in this study population.

One possible explanation for the different result observed in the present study compared to those reported previously for Dentifrice B [39, 40] may relate to differences in the sensory and aesthetic profiles of Dentifrice A versus conventional potassium nitrate-based dentifrices. Treatment with the gel-to-foam formulation has been previously shown to result in large and highly significant improvements in sensitivity relief [43]. The formulation breaks down extremely quickly during brushing, thereby facilitating rapid dispersal of the constituent ingredients throughout the mouth. In addition, patient-based in-use sensory studies (GSK-unpublished data) indicate a strong preference for the gel-to-foam formulation compared to regular desensitising dentifrice formulations. This may be expected to promote greater compliance both in terms of frequency and duration of brushing.

The prevalence of dentine hypersensitivity has been studied in a variety of different populations in a number of different countries across the world. Direct comparison of the different epidemiological studies is often confounded by the use of different clinical methodologies and definitions. The lack of homogeneity in reporting dentine hypersensitivity does make characterising the size and progression of the condition a challenge. In this respect, consensus guidelines defining the most suitable epidemiological approach to take for assessing dentine hypersensitivity prevalence would be welcome. However, what is not in dispute is that millions of people are affected by dentine hypersensitivity and that for a number of patients the severity of the problem is such that it significantly impacts on their quality of life [48]. Many more people are irritated and inconvenienced by their personal experience of dentine hypersensitivity. It may therefore be speculated that the prevalence of dentine hypersensitivity will rise as lifespans increase and people retain their dentition for longer. Prevalence levels may also be expected to increase in developing countries where increased affluence translates into behavioural adaptations that enhance susceptibility. Despite the widespread incidence of dentine hypersensitivity, it is striking that the percentage of sufferers seeking clinical intervention, and/or using otherwise available desensitising treatments such as dentifrices, are comparatively small [4]. In the latter case, there is anecdotal evidence to suggest that the negative organoleptics associated with the desensitising agent may be responsible for poor uptake and compliance. Formulation strategies that improve product tolerability may promote their continued prescribed use, and potentially enhance therapeutic outcomes.

In conclusion, this study demonstrates that the two study groups using either a potassium-based gel-to-foam dentifrice (Sensodyne Multi Action Iso-Active) or an occlusion-based dentifrice (Colgate Sensitive Pro-Relief) over an 8 week duration experienced statistically significant reductions in self- or examiner-based dentine hypersensitivity versus baseline, however no differences in levels of hypersensitivity were observable between the two groups at any timepoint.

## Figures and Tables

**Table 1:** Summary of change from baseline in efficacy measures within each treatment group; Dentifrice A = Sensodyne Multi Action Iso-Active, Dentifrice B = Colgate Sensitive Pro-Relief. *From ANCOVA model with the following factors: treatment, baseline VAS stratification value (for Schiff and Tactile analyses), number of air sensitive teeth (covariate), and baseline (covariate). ^a^Negative values favour Dentifrice A. ^b^Positive values favour Dentifrice A. Baseline values are raw means (SD).

Time point	Sensitivity parameter	Dentifrice A (*N* = 55)		Dentifrice B (*N* = 54)	
		Adjusted mean (95% CI) *P* value*	% Change from baseline	Adjusted mean (95% CI) *P* value*	% Change from baseline
Baseline	VAS	74.3 (16.31)	—	74.2 (17.36)	—
	Schiff	2.6 (0.40)	—	2.5 (0.45)	—
	Tactile	13.0 (4.47)	—	12.9 (4.19)	—

Week 1	VAS^a^	−8.4 (−13.5, −3.4)	11	−6.7 (−11.9, −1.5)	9
		0.0013		0.0115	
	Schiff^a^	−0.6 (−0.8, −0.4)	24	−0.6 (−0.8, −0.4)	25
		<0.0001		<0.0001	
	Tactile^b^	4.9 (1.4, 8.3)	38	4.4 (0.9, 7.9)	34
		0.0059		0.0137	

Week 2	VAS^a^	−20.5 (−26.7, −14.2)	28	−16.4 (−22.8, −10.1)	22
		<0.0001		<0.0001	
	Schiff^a^	−0.9 (−1.1, −0.8)	37	−0.9 (−1.1, −0.7)	35
		<0.0001		<0.0001	
	Tactile^b^	6.7 (2.7, 10.6)	51	6.9 (2.8, 10.9)	53
		0.0012		0.0011	

Week 4	VAS^a^	−24.3 (−31.6, −17.0)	33	−20.5 (−28.0, −13.1)	28
		<0.0001		<0.0001	
	Schiff^a^	−1.2 (−1.4, −1.0)	46	−1.1 (−1.3, −0.8)	42
		<0.0001		<0.0001	
	Tactile^b^	15.5 (10.4, 20.6) <0.0001	119	13.3 (8.1, 18.6) <0.0001	104

Week 8	VAS^a^	−36.6 (−44.5, −28.6)	49	−33.2 (−41.6, −24.8)	45
		<0.0001		<0.0001	
	Schiff^a^	−1.6 (−1.8, −1.4)	61	−1.5 (−1.7, −1.3)	61
		<0.0001		<0.0001	
	Tactile^b^	22.1 (16.9, 27.2)	170	20.2 (14.8, 25.6)	157
		<0.0001		<0.0001	

**Table 2:** Treatment group comparisons (ITT population) for Dentifrice A (Sensodyne Multi Action Iso-Active) versus Dentifrice B (Colgate Sensitive Pro-Relief). *From ANCOVA model with the following factors: treatment, baseline VAS stratification value (for Schiff and Tactile analyses), number of air sensitive teeth (covariate), and baseline (covariate). ^a^Calculated as (adjusted mean difference/adjusted mean for Dentifrice B) × 100. ^b^Negative values favour Dentifrice A; ^c^positive values favour Dentifrice A.

Time point	Sensitivity parameter	Difference^a^ (adjusted mean)*	% Difference^a^	95% CI*		*P* value*
				Lower	Upper
Week 1	VAS^b^	−1.7	25	−7.0	3.6	0.5263
	Schiff^b^	0.0	−1	−0.2	0.2	0.9553
	Tactile^c^	0.5	10	−3.0	3.9	0.7947

Week 2	VAS^b^	−4.0	25	−10.6	2.5	0.2215
	Schiff^b^	−0.1	7	−0.2	0.1	0.5453
	Tactile^c^	−0.2	−3	−4.2	3.8	0.9232

Week 4	VAS^b^	−3.8	18	−11.4	3.9	0.3310
	Schiff^b^	−0.1	13	−0.3	0.1	0.1695
	Tactile^c^	2.1	16	−3.0	7.3	0.4130

Week 8	VAS^b^	−3.4	10	−11.7	4.9	0.4226
	Schiff^b^	0.0	2	−0.2	0.2	0.7588
	Tactile^c^	1.9	9	−3.2	7.0	0.4619

**Table 3:** Number of subjects with reduction in sensitivity (ITT population); Dentifrice A = Sensodyne Multi Action Iso-Active, Dentifrice B = Colgate Sensitive Pro-Relief. Reduction in sensitivity defined as Schiff score decrease of at least 0.5 units; VAS score decrease of at least 10 mm; tactile pain threshold increase of at least 10 g. Percentages calculated from all subjects providing eligible data.

Time point	Sensitivity parameter	Dentifrice A (*N* = 55)			Dentifrice B (*N* = 54)		
		*n*	*N*	%	*n*	*N*	%
Week 1	VAS	22	55	40	20	54	37
	Schiff	39	55	71	33	54	61
	Tactile	16	55	29	15	54	28

Week 2	VAS	40	54	74	34	53	64
	Schiff	46	54	85	44	53	83
	Tactile	18	54	33	25	53	47

Week 4	VAS	46	55	84	37	52	71
	Schiff	50	55	91	44	52	85
	Tactile	39	55	71	32	52	62

Week 8	VAS	50	54	93	44	50	88
	Schiff	53	54	98	49	50	98
	Tactile	47	54	87	42	50	84
